# Immobilization of Lipases on Alkyl Silane Modified Magnetic Nanoparticles: Effect of Alkyl Chain Length on Enzyme Activity

**DOI:** 10.1371/journal.pone.0043478

**Published:** 2012-08-30

**Authors:** Jiqian Wang, Gang Meng, Kai Tao, Min Feng, Xiubo Zhao, Zhen Li, Hai Xu, Daohong Xia, Jian R. Lu

**Affiliations:** 1 State Key Laboratory of Heavy Oil Processing and Centre for Bioengineering and Biotechnology, China University of Petroleum (East China), Qingdao, China; 2 Biological Physics Laboratory, School of Physics and Astronomy, University of Manchester, Manchester, United Kingdom; 3 ARC Centre of Excellence for Functional Nanomaterials, Australian Institute for Bioengineering and Nanotechnology, The University of Queensland, Australia; Instituto de Engenharia Biomédica, University of Porto, Portugal

## Abstract

**Background:**

Biocatalytic processes often require a full recycling of biocatalysts to optimize economic benefits and minimize waste disposal. Immobilization of biocatalysts onto particulate carriers has been widely explored as an option to meet these requirements. However, surface properties often affect the amount of biocatalysts immobilized, their bioactivity and stability, hampering their wide applications. The aim of this work is to explore how immobilization of lipases onto magnetite nanoparticles affects their biocatalytic performance under carefully controlled surface modification.

**Methodology/Principal Findings:**

Magnetite nanoparticles, prepared through a co-precipitation method, were coated with alkyl silanes of different alkyl chain lengths to modulate their surface hydrophobicity. *Candida rugosa* lipase was then directly immobilized onto the modified nanoparticles through hydrophobic interaction. Enzyme activity was assessed by catalytic hydrolysis of *p*-nitrophenyl acetate. The activity of immobilized lipases was found to increase with increasing chain length of the alkyl silane. Furthermore, the catalytic activities of lipases immobilized on trimethoxyl octadecyl silane (C18) modified Fe_3_O_4_ were a factor of 2 or more than the values reported from other surface immobilized systems. After 7 recycles, the activities of the lipases immobilized on C18 modified nanoparticles retained 65%, indicating significant enhancement of stability as well through hydrophobic interaction. Lipase immobilized magnetic nanoparticles facilitated easy separation and recycling with high activity retaining.

**Conclusions/Significance:**

The activity of immobilized lipases increased with increasing alkyl chain length of the alkyl trimethoxy silanes used in the surface modification of magnetite nanoparticles. Lipase stability was also improved through hydrophobic interaction. Alkyl silane modified magnetite nanoparticles are thus highly attractive carriers for enzyme immobilization enabling efficient enzyme recovery and recycling.

## Introduction

Lipases are an important group of enzymes that offer powerful catalytic capability in various biocatalytic processes including hydrolysis, aminolysis, transamidation, esterification, and transesterification [1]–[4]. A common challenge in the industrial utilization of lipases and related enzymes lies in the control of catalytic activity. In a free form (e.g., when dissolved in an aqueous solution) or adsorbed on a hydrophilic surface where interaction with solvent or surface is weak, lipase mainly adopts a closed form, with weak or low activity. In contrast, when adsorbed on a hydrophobic oil/water interface, strong interaction causes changes in molecular structure, resulting in a significant increase in activity associated with its adoption of the open form [5]–[7]. Adsorption of lipases or related enzymes onto hydrophobic solid surface can induce similar structural changes with subsequent enhancement of bioactivities [4], [8]–[15]. However, there is currently a lack of correlation between the effect of surface hydrophobicity, i.e. the alkyl chain length of the modifying molecules, and catalytic activity.

Another challenge lies in the separation and recycling of enzymes while maintaining their activities. In order to simplify separation processes, enzymes are mostly immobilized onto different carriers including microporous agarose beads [8], epoxy resins [9], polypropylenes [16], copolymer spheres [17], silica particles [18], [19], aluminum silicates [20], and magnetic iron oxide nanoparticles [10]–[11], [21]–[25]. Magnetic nanoparticles are attractive because of (1) a higher specific surface area for loading a larger amount of lipase, (2) a lower mass transfer resistance for reacting substrates, (3) nontoxicity and biocompatibility, and (4) ease of separation from a reaction mixture by the application of a magnetic field. However, it is difficult to directly immobilize enzymes onto the surface of pristine magnetite nanoparticles and maintain high catalytic activities. The surface modification of magnetite nanoparticles has therefore become an attractive method for realizing their applications.

**Figure 1:**
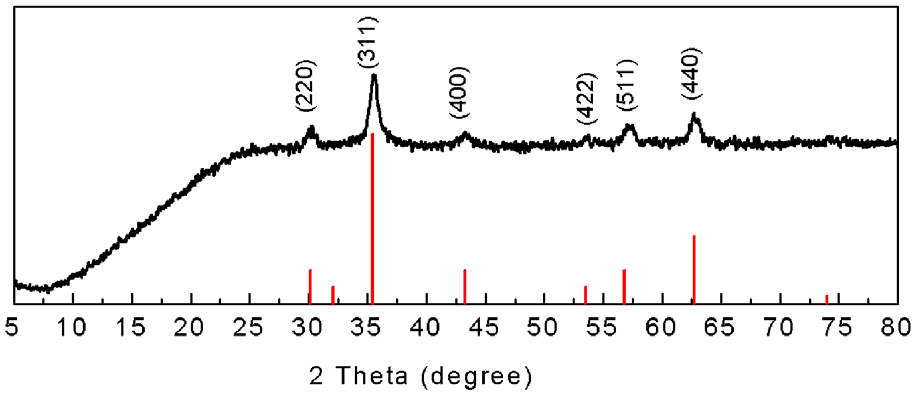
XRD pattern of Fe_3_O_4_ nanoparticles. Red lines indicates the stick pattern of JCPDS card # 3-0863 (magnetite).

Several approaches can be deployed to modify magnetic nanoparticles. The surface Fe-OH groups of hematite or magnetite can be used as modification or adsorption sites [26], [27]. Amphiphiles, such as oleic acid [28] and sodium dodecyl sulfate (SDS) [11], can be coated on the magnetite nanoparticles to create a hydrophobic surface. However, silane derivatives have a number of advantages, including (1) better structural stability through surface chemical grafting by silanization, (2) effective and efficient tuning of interfacial hydrophilicity and hydrophobicity for different applications by selection of the silane precursors, (3) attachment of various functional groups such as -NH_2_, -SH, and -COOH for further functionalization, (4) protection of magnetic particles from oxidation by coating chemically inert silica without affecting their magnetic properties [29]–[31].

In this study, we describe a simple and cost-effective method to immobilize lipase on alkyl silane coated magnetite nanoparticles through hydrophobic interaction, followed by demonstration of catalyst recycling and assessment of enzymatic activities. The results indicate that the immobilized lipase is recyclable for multiple uses while possessing high activity and stability.

**Figure 2:**
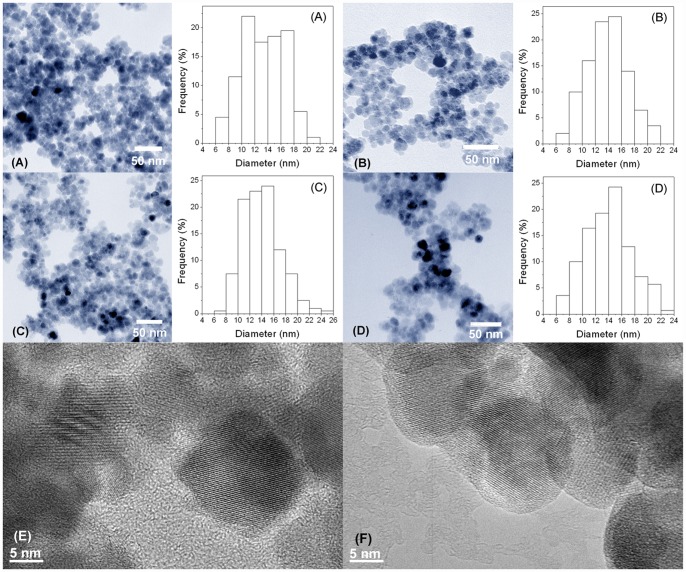
TEM images and the size distributions of nanoparticles. Fe_3_O_4_ (A), C3-Fe_3_O_4_ (B), C8-Fe_3_O_4_ (C) and C18-Fe_3_O_4_ (D) nanoparticles, and high resolution TEM images of Fe_3_O_4_ (E) and C18-Fe_3_O_4_ (F) nanoparticles.

**Table 1:** Zeta potential of magnetite nanoparticles and alkyl silane coated magnetite nanoparticles dispersed in aqueous solution at pH 7.4.

Sample	Fe_3_O_4_	C3-Fe_3_O_4_	C8-Fe_3_O_4_	C18-Fe_3_O_4_
ζ-potential/mV	11.9±0.9	5.6±1.0	7.2±0.5	–0.6±0.4

## Materials and Methods

### Chemicals

Analytical grade ferrous chloride tetrahydrate (FeCl_2_·4H_2_O) and ferric chloride hexahydrate (FeCl_3_·6H_2_O) were purchased from Tianjin Kermel Chemical Reagent Co. Trimethoxy propyl silane (98%), trimethoxy octyl silane (96%), trimethoxy octadecyl silane (>90%), *p*-nitrophenyl acetate (≥98%), and *Candida rugosa* lipase were obtained from Sigma-Aldrich. Ammonium hydroxide (25%), hydrochloric acid (37%), potassium bromide (specpure grade), sodium phosphate monobasic and dibasic (NaH_2_PO_4_ and Na_2_HPO_4_, analytical grade), and ethanol were purchased from Sinopharm Chemical Reagent Co. Ltd. All reagents were used as received without further purification. Milli-Q water was deoxygenated by boiling for several minutes and cooling down before use.

### Synthesis of Magnetite Nanoparticle and Surface Modification

Magnetite nanoparticles were made through the co-precipitation method [29], [30], [32], [33]. Typically, aqueous solutions of FeCl_3_ (0.6 M) and FeCl_2_ (0.3 M) were mixed at a ratio of 2∶1. Aqueous ammonia (3 M) was dropped into the mixture solution with vigorous stirring under N_2_ to raise the pH to 9, followed by stirring the solution at 40°C for 30 min. The mixture was then heated to 80°C and stirred for another 30 min. The magnetite particles so obtained were separated by magnetic decantation and washed immediately with deoxygenated water to neutral pH. The magnetite nanoparticles were then dried at room temperature under vacuum and ready for surface modification treatments.

**Figure 3:**
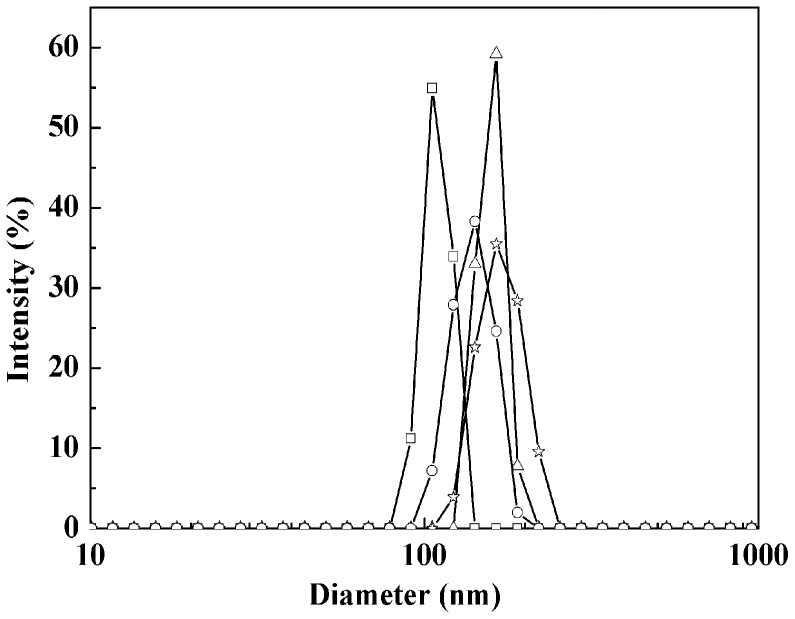
DLS hydrodynamic diameter distributions of nanoparticles. Fe_3_O_4_ (square), C3-Fe_3_O_4_ (circle), C8-Fe_3_O_4_ (triangle) and C18-Fe_3_O_4_ (star).

1.5 g magnetite nanoparticles were redispersed in 150 ml ethanol through ultrasonication. 1 ml deoxygenated water and 750 µl of an alkyl trimethoxy silane (trimethoxy propyl silane, trimethoxy octyl silane, and trimethoxy octadecyl silane, abbreviated to C3, C8, C18, respectively) were added to the nanoparticle suspension. The suspension was stirred vigorously for 10 h at 30°C. Then the nanoparticles were collected by a magnet, and washed thoroughly with ethanol to remove the excess alkyl trimethoxy silane and dried at room temperature under vacuum. Finally, the modified magnetite nanoparticles (i.e. C3-Fe_3_O_4_, C8-Fe_3_O_4_, and C18-Fe_3_O_4_) were stored under N_2_. The scheme of surface modification of the magnetite nanoparticles with silanes is shown in Figure S1.

### Characterization of Magnetite Nanoparticles

X-ray powder diffraction (XRD) analysis of the magnetite nanoparticles was carried out by a PANalytical X’Pert PRO diffractometer with Cu-Kα radiation operated at 40 kV and 40 mA. The scan range of 2θ was from 5° to 80°. Surface bonding and functional groupings of the nanoparticles were studied by Fourier transform infrared (FTIR) spectroscopy using a Nicolet 6700 FTIR spectrometer (Thermo Scientific, US). The hydrodynamic radius and ζ-potential of the nanoparticles was determined with the Nano ZS zetasizer (Malvern Instruments, UK). The nanoparticles were dispersed in aqueous solution at pH 7.4, and their ζ-potential was determined at 25±0.1°C at a wavelength of 633 nm. The ζ-potential is the average of at least 5 runs for each sample. The size and morphology of the nanoparticles were observed by a transmission electron microscope (TEM) (JEOL-1200EX, Japan) with an accelerating voltage of 120 kV. High resolution TEM images of Fe_3_O_4_ and C18-Fe_3_O_4_ were obtained from a JEM-2100 electron microscope (JEOL, Japan).

**Figure 4:**
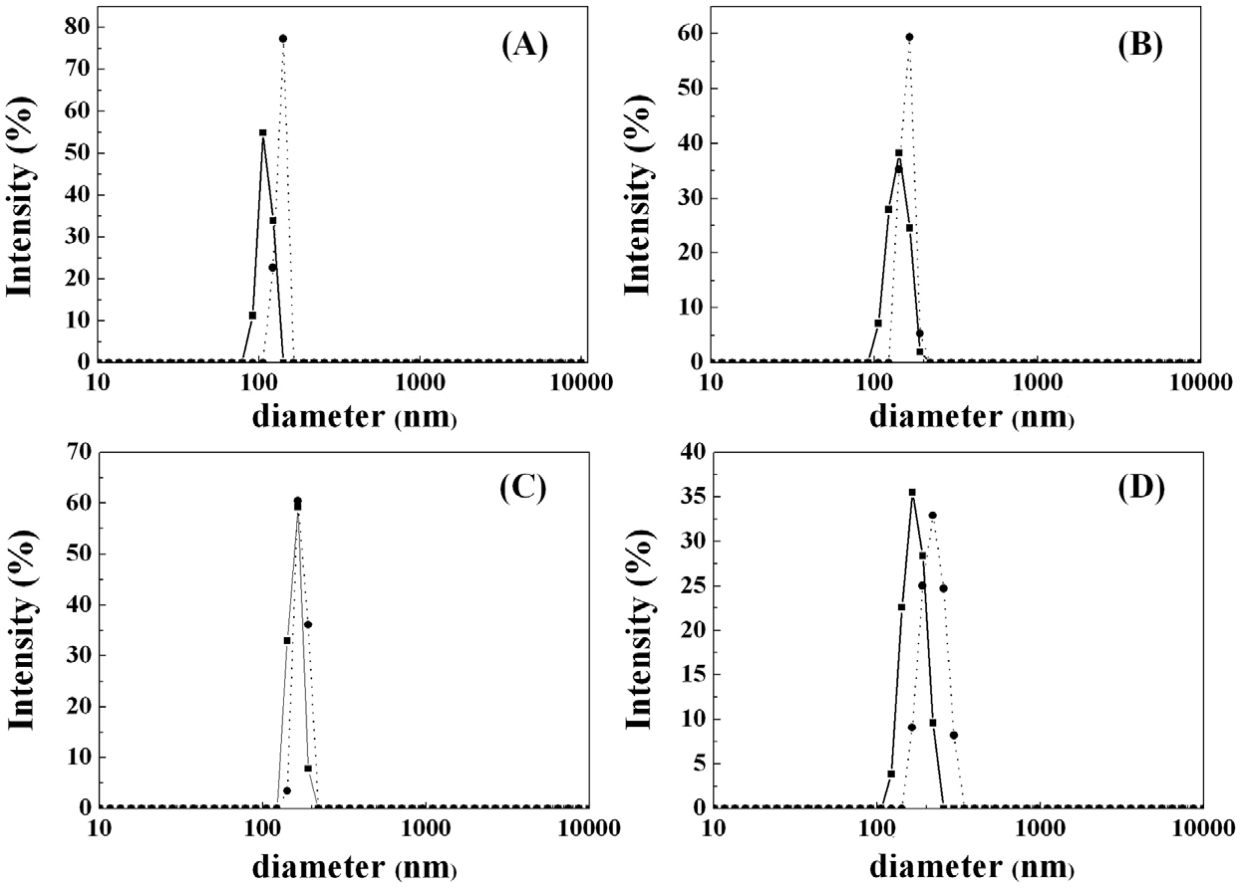
DLS size distributions of the nanoparticles before (solid line) and after (dot line) lipase immobilization. A, Fe_3_O_4_; B, C3-Fe_3_O_4_; C, C8-Fe_3_O_4_; and D, C18-Fe_3_O_4_.

**Figure 5:**
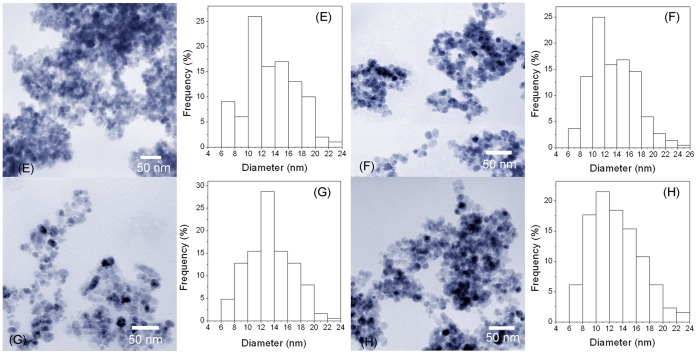
TEM images of Fe_3_O_4_ (E), C3-Fe_3_O_4_ (F), C8-Fe_3_O_4_ (G), and C18-Fe_3_O_4_ (H) nanoparticles immobilized with lipase.

### Lipase Immobilization and Activity Assay

30 mg of unmodified or modified magnetite nanoparticles (C3-Fe_3_O_4_, C8-Fe_3_O_4_, or C18-Fe_3_O_4_) were well dispersed in 5 ml of phosphate buffered saline (PBS) solution (pH = 7.4) through 5 min sonication. The suspension was then dropped into 15 mL of *Candida rugosa* lipase PBS solution of a certain concentration. The mixture was gently shaken at room temperature for 24 hr to immobilize lipase onto the nanoparticles. The lipase immobilized nanoparticles were then separated by a magnet, and washed by PBS several times before being redispersed into 6 ml of PBS solution to get a lipase immobilized nanoparticle stock solution at the concentration of 5 mg/mL for the determination of lipase activity. The amount of residual lipase in supernatant and washed solution was determined by the adsorption at 280 nm on a UV-Vis spectrometer (Shimadzu 2450, Japan) [34]. The amount of lipase bound to magnetic nanoparticles was calculated by subtracting the amount of residual lipase in solution from the amount of lipase in the original solution.

The enzymatic activity of the immobilized lipase was determined by the following procedure. 500 µl of 0.1 M *p*-nitrophenyl acetate (*p*-NPA) solution was added into 100 ml PBS, and then 2 ml of immobilized lipase suspension of 5 mg/ml was mixed with the *p*-NPA solution. The hydrolysis reaction of *p*-NPA was carried out at 37°C with gentle stirring for 30 min. The immobilized lipase and magnetic nanoparticles fell to the bottom of the flask and the amount of released *p*-nitrophenol was monitored by absorption at 405 nm using UV-vis spectroscopy [23], [35]. The immobilized lipase was separated for reuse. One unit (U) of enzyme activity is defined as the amount of enzyme necessary to hydrolyze *p*-NPA to release 1.0 µmol of *p*-nitrophenol in 1 min under the assay conditions. All samples were tested at least three times, and the arithmetic means and standard deviations were calculated.

**Figure 6:**
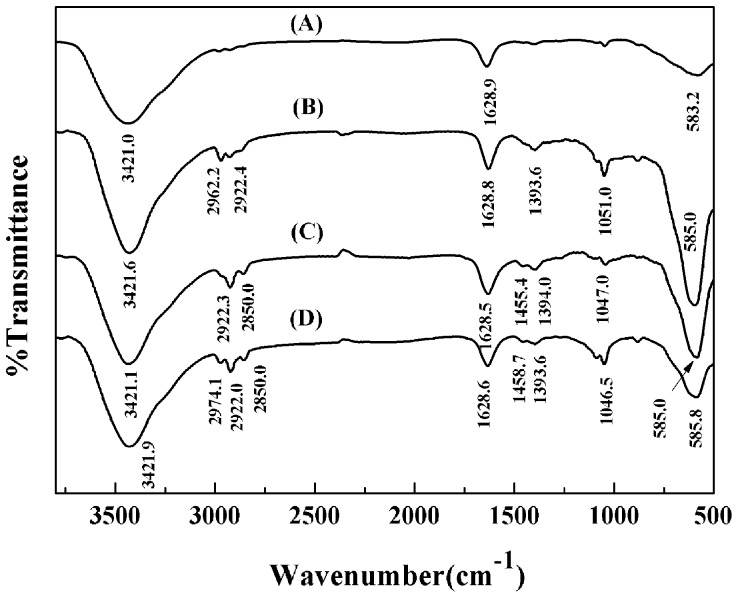
FTIR spectra of Fe_3_O_4_ (A), C3-Fe_3_O_4_ (B), C8-Fe_3_O_4_ (C), and C18-Fe_3_O_4_ (D) nanoparticles.

## Results and Discussion

### Properties of Modified Magnetite Nanoparticles

The XRD pattern of magnetite nanoparticles shown in Figure 1 indicates the cubic inverse spinel structure of Fe_3_O_4_ according to JCPDS card 00-003-0863 [30], [33], [36]. Three trimethoxy silanes with different alkyl chain lengths, trimethoxy propyl silane (C3), trimethoxy octyl silane (C8), and trimethoxy octadecyl silane (C18), were selected to modify Fe_3_O_4_ nanoparticles to obtain hydrophobic surfaces. Figure 2 shows the TEM images and the size distributions of the Fe_3_O_4_, C3-Fe_3_O_4_, C8-Fe_3_O_4_, and C18-Fe_3_O_4_ nanoparticles following surface modification. Most of the nanoparticles are quasi-spherical, and their diameters are 13.5±3.2 nm, 13.9±3.1 nm, 14.0±3.2 nm, and 14.0±3.6 nm, respectively, indicating little or no difference in particle size before and after modification. However, as shown in Table 1, the zeta potential of the magnetite particles significantly decreased after coating with alkyl silane compounds, indicating different extent of shielding of the bare oxide surface. The surface of magnetite is amphoteric, and the surface charges originate from the protonation or deprotonation of surface Fe-OH according to the pH environment. Changes in zeta potential indicated that the magnetite nanoparticles were coated with silane groups successfully, consistent with the IR results. Because of the steric hindrance of alkyl silanes, not all the hydroxyl groups on the particle surface reacted with alkyl trimethoxy silanes. Therefore, the zeta potentials were no-zero from C3-Fe_3_O_4_ and C8-Fe_3_O_4_ as expected. However, if the alkyl chains are very long (e.g. C18-Fe_3_O_4_), they can provide the nanoparticles with a strong hydrophobic layer to prevent proton diffusion to the Fe_3_O_4_ surface, resulting in almost zero zeta potential of C18- Fe_3_O_4_. Hence, the non-zero zeta potentials from C3-Fe_3_O_4_ and C8-Fe_3_O_4_ arose from a partial silane neutralization and weak alkyl chain coverage, leaving surface defects for surface proton diffusion. The TEM images and histograms show that these surface modifications did not lead to any major aggregation of the nanoparticles either. From the high resolution TEM image (Figure 2-E), a very thin layer was observed on the outside surface region of the C18-Fe_3_O_4_ nanoparticles (Figure 2-F) associated with the hydrolysis and condensation of alkyl trimethoxy silanes.

**Figure 7:**
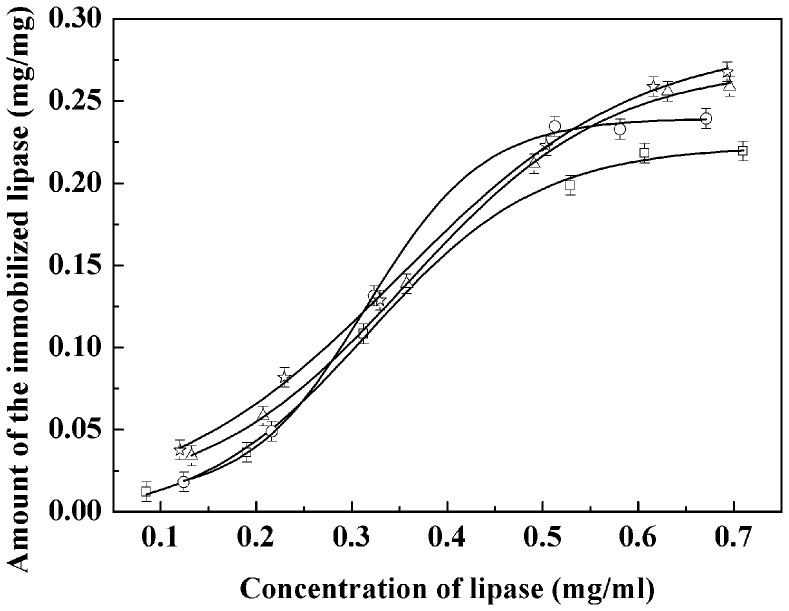
The amount of the lipase immobilized onto different nanoparticles. Fe_3_O_4_ (square), C3-Fe_3_O_4_ (circle), C8-Fe_3_O_4_ (triangle), and C18-Fe_3_O_4_ (star).

A useful observation was the effective dispersion of the coated nanoparticles in aqueous solution, given a varying degree of surface hydrophobicity after different silane coating. The hydrodynamic particle sizes of these nanoparticles have also been determined by dynamic light scattering (DLS), with the results shown in Figure 3. The hydrodynamic diameters are overall larger than the values as determined by TEM. Such large size differences indicate the formation of aggregates of these nanoparticles in aqueous solution. It is interesting to note that all particles have their peak sizes around 100 nm. Within the experimental error, however, Figure 3 shows a small but clear increase of aggregate size with the chain length of surface modification, an effect arising from increasing surface hydrophobicity. The hydrodynamic size order is: C18-Fe_3_O_4_> C8-Fe_3_O_4_> C3-Fe_3_O_4_> Fe_3_O_4_, Figure 3 also reveals similar unimodal aggregate size distributions.

**Figure 8:**
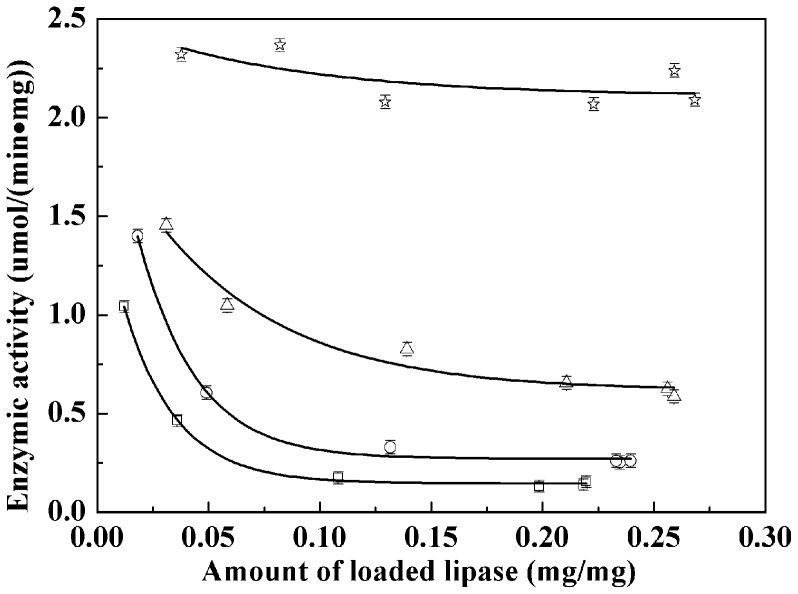
Enzyme activity of the immobilized lipase on different nanoparticles. Fe_3_O_4_ (square), C3-Fe_3_O_4_ (circle), C8-Fe_3_O_4_ (triangle), and C18-Fe_3_O_4_ (star).

Adsorption of lipase alters surface amphiphilicity and may change the nature of nanoparticle aggregation. Figure 4 shows the variation of hydrodynamic aggregate sizes before and after lipase immobilization. The lipase immobilized Fe_3_O_4_ nanoparticle aggregates have slight size increase in aqueous solution according to the DLS results. This trend is the same for all nanoparticle systems. However, the TEM results (Figure 5) measured from high vacuum conditions show that the particle sizes are almost the same as those without surface silane coating or lipase immobilization, confirming that the TEM sizes only provide a good measure of the individual nanoparticle cores and have no relevance to solution behavior before and after lipase immobilization [28], [37].

**Figure 9:**
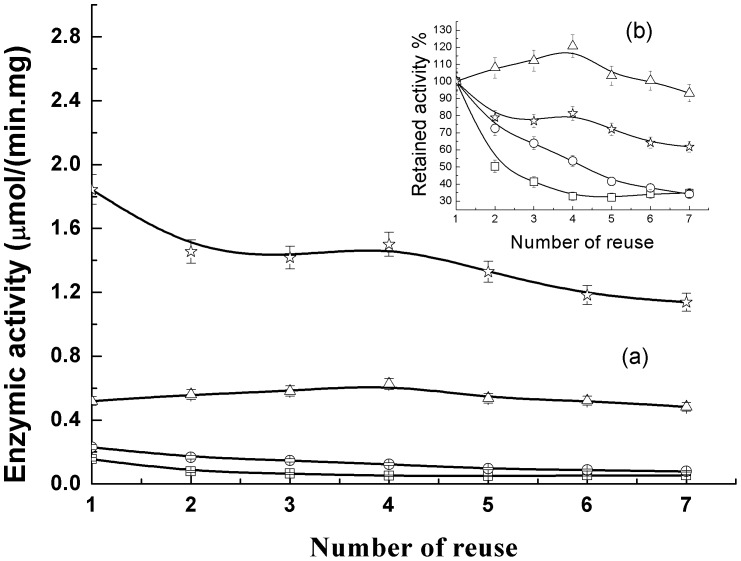
Reusability of the immobilized lipase on different nanoparticles. Fe_3_O_4_ (square), C3-Fe_3_O_4_ (circle), C8-Fe_3_O_4_ (triangle), and C18-Fe_3_O_4_ (star).

To verify the binding of silanes on the surface of nanoparticles, FTIR spectra of Fe_3_O_4_ nanoparticles before and after alkyl silane coating were collected, with the results shown in Figure 6. Peaks at ∼2974, ∼2922, and ∼2850 cm^-1^ originated from the stretching vibrations of C–H bonds in alkyl chains, and peaks at ∼1455 and ∼1395 cm^-1^ are due to the in-plane bending vibrations of -CH_3_ and -CH_2_-. The intensity and position of these bands are slightly different because of the length differences of the alkyl chains. The strong absorption peak at ∼583 cm^-1^ is the characteristic band of the Fe-O stretching vibrations of Fe_3_O_4_ nanoparticles. The Fe-O stretching vibration band of the bulk magnetite is usually at ∼570 cm^-1^, and the band shifts to high wave numbers because of the finite size of nanoparticles [29]. Under the influence of broken surface bonds, the delocalized electrons on the particle surface rearrange to give the blue-shift of the absorption bands [29]. Compared with the uncoated magnetite nanoparticles, the Fe-O absorption bands of Fe_3_O_4_ nanoparticles coated with alkyl silanes are shifted to a higher wavenumber of ∼585 cm^-1^, due to the formation of Fe-O-Si bonds on the surface of the coated nanoparticles. Since -Si(O-)_3_- is more electronegative than H, it leads to an increase of force constant of Fe-O bonds and the consequent shift of the peak position [38]. The weak bands at ∼1050 cm^-1^ correspond to Fe-O-Si stretching vibrations, showing that alkyl silanes are successfully grafted on Fe_3_O_4_ nanoparticles. Two peaks at ∼3400 and ∼1630 cm^-1^ are attributed to the stretching and bending vibrations of the O-H band from residual water in the samples.

### Immobilization Efficiency of Lipase on Magnetite Nanoparticles

Figure 7 shows the relationship between the concentration of lipase solution and the immobilized amount on the surface of modified and unmodified magnetite nanoparticles. It was found that with increasing lipase concentration, the amount of immobilized lipase increased and reached a saturated amount of about 0.25 mg lipase per milligram magnetite nanoparticles when the lipase concentration was above 0.55 mg/ml. The amount of lipase on alkyl silane coated nanoparticles is larger than that on uncoated nanoparticles, but there is no distinct difference among the three differently modified nanoparticles, i.e. between C3-Fe_3_O_4_, C8-Fe_3_O_4_, and C18-Fe_3_O_4_. This observation simply shows that the amount of lipase immobilization is not affected hugely by different surface hydrophobicity under these experimental conditions. In brief, whilst there is a clear influence between surfaces of different chemical nature (hydrophilic bare nanoparticle surface versus coated hydrophobic surface), the effect of different alkyl chain lengths appears to be small as far as the surface loading is concerned.

The molecular weight of *Candida rugosa* lipase is 57 kDa. Its molecular dimensions are a = 64.9, b = 97.5, c = 175.6 Å (orthorhombic crystal, space group *C222*_1_) [7]. If we postulate that the shape of lipase is cubic, the surface areas of each face are 0.63×10^–12^, 1.13×10^–12^, and 1.71×10^–12^ cm^2^. Assuming that magnetite nanoparticles are spherical and monodisperse (d = 14 nm), their specific surface area is 840 cm^2^/mg. The theoretical maximum immobilized amount of one monolayer of lipase is 0.13 mg per mg of nanoparticles. Our experimental immobilized amount is about 0.20 mg lipase per mg of nanoparticles, indicating that the immobilization is very effective and that the physical adsorption on the Fe_3_O_4_ nanoparticles must have resulted in high surface packing involving multilayer formation. This verdict is also reached if one tries to estimate surface packing from the hydrated solution dimensions of lipase from a recent small angle neutron scattering (SANS) study by Sate et al. [39].

### Effect of Surface Modification on the Activity of Immobilized Lipase

The relationship between the hydrolytic activity and the amount of immobilized lipase is shown in Fig 8. It can be seen that lipases immobilized on alkyl trimethoxy silane coated Fe_3_O_4_ nanoparticles (C3- Fe_3_O_4_, C8-Fe_3_O_4_, and C18-Fe_3_O_4_) exhibit higher catalytic activities than those on uncoated Fe_3_O_4_. Furthermore, the lipases immobilized on C18- Fe_3_O_4_ show the highest activity of 2.1×10^−6^ mol/min⋅mg. Previous studies [23]–[24] showed that the activities of free lipases and lipases covalently bound on Fe_3_O_4_ magnetic nanoparticles are 0.76×10^−6^ and 1.07×10^−6^ mol/min⋅mg in the hydrolysis of 4-nitrophenyl palmitate (p-NPP). Our lipase activities showed an increase by a factor of 2 or more, which must be attributed to the increased surface hydrophobicity.

A polypeptide lid covers the catalytic active center of lipase and the external surface of the lid is hydrophilic, while its internal surface, pointing towards the catalytic center, is hydrophobic [6], [7]. When the lipase is immobilized on a hydrophobic surface, the hydrophobic interaction between the enzyme and the surface makes it structurally deformed so that the catalytic active center becomes available for substrate to access and interact. Thus, the activities of lipases immobilized on hydrophobic supports, such as octadecyl-sepabeads, octyl-agarose, oleic acid-coated magnetite, carboxylic surfactant grafted zirconia nanoparticles, SDS-bound nano-sized magnetite particles, are enhanced [4], [8]–[14]. In contrast, if lipases are immobilized on hydrophilic surfaces, the hydrophilic interaction between the surface and the external surface of the lid does not lead to lid opening. As a result, enzymatic activities remain low [4], [16]. Comparing the activities of lipases loaded on alkyl trimethoxysilane modified Fe_3_O_4_ nanoparticles, we found that the effect of alkyl chain length on lipase activity is in the order of C18-Fe_3_O_4_> C8-Fe_3_O_4_> C3-Fe_3_O_4_. This means that lipase activity increases with the hydrophobicity of the support surface. It should be noted that the surface zeta potentials of C8-Fe_3_O_4_ and C3-Fe_3_O_4_ were non-zero whilst that of C18-Fe_3_O_4_ was close to zero. Different surface residual charges could affect the alignment of lipases once immobilized, leading to the different accessibility of the active site. Thus, the lack of surface charge in the case of C18-Fe_3_O_4_ could also contribute to the activity enhancement, but there is no experimental evidence to prove this in the context of this study.

In addition, Figure 8 shows that the activity decreases with an increasing load of lipase for all four kinds of nanoparticles. This observation can be explained by the steric hindrance which becomes stronger with an increasing adsorbed amount of lipase. The lipase surface coverage dependent activity becomes weak when surface hydrophobicity increases. In the case of C18-Fe_3_O_4_, the catalytic activity of the immobilized lipase is always above 2.0×10^−6^ mol/min⋅mg over the whole range of the adsorbed amount studied. This indicates that the steric effect becomes less significant when interfacial activation is induced by higher surface hydrophobicity.

Different surface chemistries must affect the secondary structures of lipases immobilized. Solanki and Gupta have recently examined the impact of surface immobilization by circular dichroism (CD), but the interferences arising from nanoparticles undermined the quality of CD spectra, making it difficult to make useful inference of structural variations [40]. FTIR is another method to determine the secondary structures with the amide I absorption peaks between 1600 and 1700 cm^-1^. However, there’s also a peak of Fe_3_O_4_ nanoparticles at 1628 cm^-1^ as shown in Fig 6, again making it difficult to infer the changes of secondary structures of surface immobilized lipases.

### Reusability of Immobilized Lipase

The reusability of the immobilized lipase is important for potential industrial applications. The advantage of using magnetic nanoparticles as lipase carriers is not only the enhancement of catalytic activity but also the simple separation processes for recovery and recycling. In order to investigate reusability, the lipase immobilized nanoparticles were separated using a magnet and washed with PBS after every cycle and redispersed into a fresh *p*-NPA solution for the next cycle of hydrolysis reaction. The variation of activity is shown in Figure 9. It was observed that the immobilized lipase on C18-Fe_3_O_4_ retained 65% of its initial activity after 7 cycles. The decrease of activity could result from the denaturation and leaching (detachment) of lipase molecules from nanoparticles. The results indicate that the strong hydrophobic interaction between lipase and modified Fe_3_O_4_ nanoparticles enhances both activity and stability.

### Conclusions

The immobilization of lipase onto alkyl trimethoxy silane modified magnetite nanoparticles has been studied with respect to different hydrocarbon chain lengths. Surface immobilization provided easy separation and resue of the biocatalyst. Importantly, the immobilized lipase showed improved enzymatic activities with increasing hydrocarbon chain length, with the longest hydrocarbon chain modified surface offering the highest activity. Hydrophobic interaction must have induced conformational rearrangements of the immobilized lipase molecules into more active forms. The lipase stability was also improved through hydrophobic interaction. Recycling of the lipase samples showed that lipase immobilized on magnetic nanoparticles was very stable. The catalytic activity of lipase on C18-Fe_3_O_4_ retained 65% of its starting value even after recycling 7 times. The magnetite nanoparticles modified with an alkyl trimethoxy silane of a proper alkyl length are therefore excellent candidates as efficient carriers for lipase immobilization.

## Supporting Information

Figure S1 **Modification of magnetite nanoparticles by trimethoxy alkyl silanes.**
(TIF)
