# Supplementary figures and images for: Immobilization of Lipases on Alkyl Silane Modified Magnetic Nanoparticles: Effect of Alkyl Chain Length on Enzyme Activity
Source: PLoS One. 2012 Aug 30;7(8):e43478. doi: 10.1371/journal.pone.0043478 (PMC3431390; doi:10.1371/journal.pone.0043478)

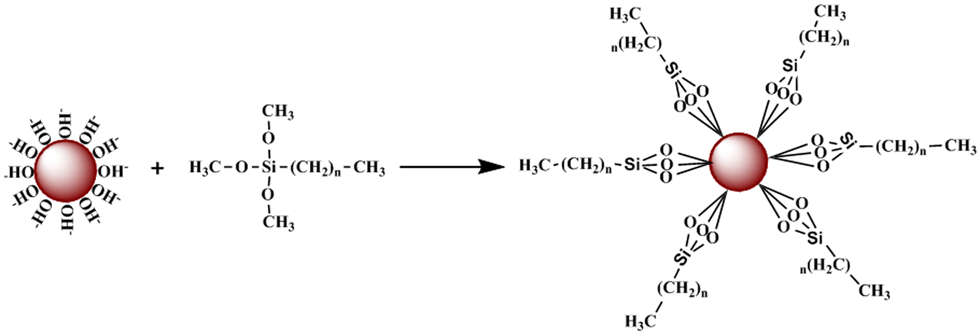

Supplement: Figure S1 — Modification of magnetite nanoparticles by trimethoxy alkyl silanes. (TIF) [file pone.0043478.s001.tif]
